# Stroke in Patients with Schistosomiasis: Review of Cases in Literature

**DOI:** 10.1155/2022/3902570

**Published:** 2022-07-25

**Authors:** Valeria Silvestri, Vivian Mushi, Billy Ngasala, Jacqueline Kihwele, Deodatus Sabas, Lorenzo Rocchi

**Affiliations:** ^1^Department of Parasitology and Medical Entomology, Muhimbili University of Health and Applied Science, P.O. Box 65011, Dar es Salaam, Tanzania; ^2^Department of Internal Medicine, Muhimbili University of Health and Applied Science, P.O. Box 65001, Dar es Salaam, Tanzania; ^3^Directorate of Library Service, Muhimbili University of Health and Allied Sciences, Dar es Salaam, Tanzania; ^4^Department of Medical Sciences and Public Health, University of Cagliari, Cagliari, Italy

## Abstract

**Introduction:**

Cerebral vascular comorbidities may occur in patients with schistosomiasis, as described in case reports. *Aim and Methods.* We have summarized general clinical and neurological features in patients with stroke associated with schistosomiasis, through a review of case reports in the literature. *Investigation Outcomes.* A total of eight case reports were retrieved. The mean age of patients was 36.42 ± 16.7 (19 to 56 years), four females, three males, and one anonymous sex. Eosinophilia was the most frequent feature at presentation, followed by cardiac abnormalities, confusion, fever, ataxia, hemiplegia, headache, urticaria, dysphasia, and memory impairment. Patients usually present with watershed infarction or intracranial vasculitis. In one case, extracranial carotid arteries presented with inflammation and stenosis. The patient's serology was positive on admission in five cases. Full neurological recovery was reported in three cases, and partial improvement in another three. In two cases, information on neurological outcomes was incomplete. Stroke in schistosomiasis can be caused by haemodynamic impairment, direct lesion to the arterial wall, vasa vasorum obliterative endarteritis, contiguity with a focus of inflamed tissue, or inflammatory intimal damage. Schistosomiasis needs to be included in the differential diagnosis of stroke in people living or coming back from endemic areas.

**Conclusions:**

Further studies addressing the noncommunicable comorbidity issues related to this condition are needed.

## 1. Introduction

First described in 1851 by the German physician Theodor Bilharz, bilharziasis (or schistosomiasis) is an infestation caused by one of six species of trematode worms of the genus *Schistosoma*, which have different geographic distributions. *S. haematobium* is the most common species, occurring in sub-Saharan Africa and the Middle East [1]. Globally, 779 million people are at risk, and about 250 million are infested by Schistosoma spp., of which 201.5 million live in Africa. According to the Global Burden of Disease Study 2016, the estimated schistosomiasis global burden is 1.9 million disability-adjusted life years (DALYs) [1]. Recently, increased population movements due to climate change are directly linked to the spread of schistosomiasis [1].

Schistosome infestation in humans occurs by contact with fresh water contaminated by free-swimming cercariae, which represents the infectious stage of schistosomes, that are released by an intermediate host snail and can penetrate the intact human skin. Acute schistosomiasis, also known as Katayama fever, occurs after a primary infestation, usually between 2 weeks and 3 months after exposure to larvae, which enter small venous or lymphatic vessels, to be later transported to their maturation site in the liver. Symptoms such as fever, myalgia, and hematuria are caused by the formation of immune complexes in response to antigens released during schistosomula migration [1]. The acute stage is followed by an established phase of the invasion, which is usually asymptomatic due to the presence of somatic stem cells that regenerate the surface tegument of adult schistosome and can bind to host antigens, thus escaping the host's immune system [1]. The third stage of infestation is caused by the deposition of schistosome eggs in blood vessels, where they actively secrete antigenic glycoproteins that facilitate their passage to the lumen of the guts or urinary bladder, with the aid of the induced inflammatory response. Inflammation may also induce the formation of granulomas and consequent entrapment of eggs in surrounding tissues.

Organ-specific morbidity, according to the infecting Schistosoma spp., can develop in chronic stages. Clinical manifestations are caused by the inflammatory response of the host to the accumulation of parasite eggs in vessels and organs. For example, the involvement of mesenteric veins and small portal branches in the liver may cause liver fibrosis and portal hypertension [2]. Urogenital schistosomiasis can develop as a consequence of pelvic venous plexus involvement; hydronephrosis and kidney insufficiency may occur in severe cases, and an association with squamous cell carcinoma of the urinary bladder has also been described [1]. Schistosome worms and eggs can sometimes locate in the spleen, lungs, skin, and central nervous system, causing site-specific manifestations. For instance, in pulmonary schistosomiasis, caused by portal-caval shunting, eggs are transported to the lung capillaries, where they induce granulomas in the perialveolar area, leading to fibrosis and potentially resulting in pulmonary hypertension and cor pulmonale [1, 3].

Vascular complications of schistosomiasis are less described than the involvement of other organs or systems. Arterial damage can occur by direct lesion of arterial walls due to obliterative endarteritis involving vasa vasorum or contiguity with a focus on inflammation in surrounding tissues [4]. The vasculitic process has been described in intra- and extracranial carotid arteries [5, 6]. Therefore, the need to include the suspicion of schistosomiasis in the differential diagnosis of stroke and cerebral vasculitis has been emphasized in literature [6], especially since the prevalence of schistosomiasis is likely to increase in the near future due to climate changes and migrations [1]. Noncommunicable diseases (NCD) have been included in sustainable development goal (SDG) target 3.4, aiming to reduce premature mortality from these conditions by one-third by 2030 [7]. Still, the lack of easy-to-consult sources of data and information in many sub-Saharan settings [8] may lead to underreporting and underestimation of many conditions that could be relevant in this specific geographic-epidemiological setting, such as stroke occurring in areas with a high incidence of schistosomiasis and associated with schistosoma infestation. This highlights the need for preliminary studies that could guide further research in this specific area, in which parasitological and neurovascular interests overlap.

## 2. Aims and Methods

Our review aims to report the cerebrovascular involvement during schistosomiasis, as well as to clarify the features of cerebrovascular involvement and the possible outcome after established neurological damage.

### 2.1. Search Strategy and Inclusion Criteria

Current literature has been reviewed consulting MEDLINE, EMBASE, and Scopus for studies involving patients with a history of schistosomiasis presenting with stroke or cerebral vasculitis, using “Schistosomiasis AND stroke” OR “Schistosomiasis AND cerebral vasculitis” as keywords. All articles published between 1980 and April 2021 and written in English and French were considered.

**Figure 1:**
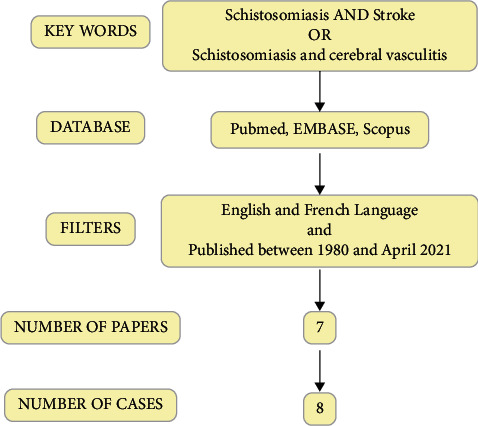
Review of literature from research strategy to number of papers included in the study.

A flow chart of the review process is provided in Figure 1.

### 2.2. Quality Assessment

Quality assessment of studies was carried out according to the checklist by Murad and colleagues [9], based on selection, ascertainment, causality, and reporting domains. More specifically, the checklist assesses for each paper availability of data from the whole investigator's experience; adequate addressing of exposure; clear report of outcome; details on differential diagnosis; description of challenges; dose-response effect description; sufficient length of follow-up; report of sufficient details to allow inference-making. Details on treatment, neurological, and general outcomes were collected, where available. Given the paucity of cases reported in the literature, the lack of details related to patients outcome was not considered an exclusion criterion.

### 2.3. Data Analysis

Due to the heterogeneity of reports, a qualitative approach was used to analyze the studies and summarize findings. Percentages and frequencies were generated for qualitative variables. Descriptive statistics (mean, standard deviation, median, and interquartile range) were used to present quantitative variables. The review of literature has been reported using a standard format [10].

## 3. Investigation Outcomes

From the literature review, we found seven papers describing eight cases (Table 1). According to Murad's checklist [9], seven out of eight case reports had a quality score between 7 and 8. The lack of follow-up was the feature of the quality checklist most frequently lacking. Incomplete differential diagnosis (or lack of diagnosis of certainty through a histology specimen) was responsible for other missing points on the checklist. The mean age of patients with schistosomiasis and stroke was 36.42 ± 16.7, ranging from 19 to 56 years. About 50% (four cases) were female and three (37.5%) were males, while in one case information on sex was missing.

### 3.1. Summary of Cases

Sarazin and colleagues reported the case of a 25-year-old female with a history of recent travel to Madagascar, presenting with fever for a week, rapidly progressive headache, limb ataxia, and memory impairment. She was diagnosed with watershed bilateral infarction. Stool positivity and seroconversion occurred only 3 weeks after neurological presentation. The case was complicated by progressive cardiomyopathy requiring surgery. No neurological recurrence occurred [11].

Sonneville and co-workers described a case of a 56-year-old woman with sudden onset of hemiplegia due to watershed infarction. Acute pericarditis and myocarditis complicated the case. The outcome was favorable with steroidal therapy, and neurological recovery was reported after 6 months [12].

Two case reports were published by Jaureguiberry. A 54-year-old man living in Mali for several years, where he had been repeatedly exposed to fresh groundwater, was medically examined due to a history of fever for 10 days, followed by acute cerebellar syndrome, hemiparesis, and confusion, with multiple cortical and subcortical infarcts caused by cerebral vasculitis. Serology and urinary positivity for the presence of schistosoma eggs was observed 4 months after the acute event when the patient still showed psychomotor slowing and insomnia [13]. The second case reported a 21-year-old male patient who bathed 1 month earlier in a lake in Mali. Four days after starting therapy with praziquantel due to a maculopapular rash and fever, he presented with confusion and anosognosia, and he was diagnosed with cerebral vasculitis. The patient had a positive serology at hospitalization, but schistosoma eggs could be detected in urine only 4 months later. The neurological features resolved after 48 hours of steroid therapy [13].

In the report by Camuset and coworkers, a 28-year-old female patient went on a mission in Burkina Faso for 1 year, where she bathed in a lake. She had a 6-month history of headaches. She was hospitalized for hemiplegia associated with an unspecified language disorder. Serology and rectal biopsy showed positivity for schistosoma. One month later, the patient presented with headache, diplopia, and VI cranial nerve palsy, with stenosis of carotid syphon. The exacerbation of vasculitis was considered due to the adverse effects of praziquantel. Minor improvement of carotid stenosis was reported at 6 months, but no details on hemiplegia were provided [6].

The case reported by Wu and colleagues lacked anagraphic details. The patient presented with acute headache and walking impairment, and multiple low densities in the alba were observed on imaging. Exposure history suggested chronic schistosomiasis [14].

Grandièr-Peréz and coworkers reported the case of a 19-year-old girl back from Kenya, where she had bathed in lake Victoria 2 weeks earlier. She presented with a 15-day history of fever and urticaria. Neurological features occurred on day 12, with the appearance of ataxia, confusion, and unilateral dysmetria. Imaging showed features of watershed infarction. Serology was positive at hospitalization, while stool and urine became positive for the presence of schistosoma eggs 4 months later. A full neurological recovery was reported 5 days after starting steroidal therapy [15].

Lastly, in the report by Nyein and colleagues, a 52-year-old patient had visited Myanmar during a schistosomiasis outbreak 1 year earlier, had swum in ponds and had eaten snails, and was hospitalized because of cognitive impairment, memory deficits, and behavioral changes, with multiple cerebral infarcts on imaging. This picture was followed, 3 weeks later, by dysarthria, hemiplegia, and facial palsy, with frontal lesions on imaging, bilateral terminal internal carotid artery narrowing, and beaded anterior cerebral artery. New frontal lesions appeared at week 9 after hospitalization. The patient had a positive serology, but no eggs were detected by optical microscopy assessment of stool. On follow-up, neurologic recovery was partial, and the patient could walk with the aid [16]. The cases are summarized in Table 1; for clarity, each case was assigned with a number, which will be used further in the text to refer to single patients. Tables 2 and 3 reports the incidence of specific neurological symptoms.

### 3.2. Schistosomiasis in Differential Diagnosis for Stroke at Younger Age

The mean age of patients was found to be 36.42 ± 16.7 years (min 19, max 56). It has been reported that only 10% to 15% of all strokes occur in adults aged 18 to 50 years and that the underlying pathophysiology and risk factors for stroke at younger age differ from those that occur in older patients. While the most common types of ischemic stroke in older adults are large artery atherosclerosis and small-vessel occlusions, these two subtypes accounting for only 10% to 20% of strokes in younger adults. In the latter group, other aetiologies/risk factors need to be considered, including the use of estrogens, pregnancy, migraine with aura, patent foramen ovale, inherited thrombophilia, acquired prothrombotic or hypercoagulable states, metabolic syndrome, carotid or vertebral artery dissection, and vasculitis [17]. Stroke secondary to diseases of parasitological interest, such as schistosomiasis, should be considered as a possible cause of stroke in the young living in endemic areas or for patients coming from these regions.

### 3.3. Pathophysiology of Stroke in Schistosomiasis

Vascular complications in schistosomiasis can be caused by several pathophysiological mechanisms, including haemodynamic impairment, direct lesion to the arterial wall (obliterative endarteritis involving vasa vasorum), inflammatory intimal damage, or direct damage due to contiguity with a focus on inflammation in surrounding tissues [4]. It is interesting to note that, apart from primary schistosoma infestation [11, 13, 15], watershed infarction and cerebral vasculitis may occur during or be a delayed manifestation of chronic infestation (as in cases 5 and 8 of the present review).

### 3.4. Egg-Induced Cerebral Vascular Damage

Egg deposition usually occurs 3–4 weeks after infestation. While 20% to 55% of excreted eggs successfully reach the urinary bladder (*S. haematobium*) or the intestine (*Schistosoma mansoni* and *Schistosoma japonicum*) to be finally excreted in the environment, the remainder can embolize at various other locations, including the nervous system, where inflammation can develop [18, 19]. In the case of neuro-Schistosomiasis, the inflammatory environment established by eggs may also exacerbate established pathology or create an environment that favors other pathogen survival, suppressing the immune responses toward viral and bacterial antigens; ineffective pathogen clearance and chronicity may occur [18].

### 3.5. Carotid Artery Involvement and Cerebral Vasculitis

Two forms of vascular changes have been described in the literature in cerebral schistosomiasis caused by *S. mansoni* infestation. The first is represented by arteritis with fibrinoid necrosis involving the arterial wall, with or without ova or granulomatous lesions. The second involves arterial wall thinning, interruption of the internal elastic membrane, aneurysmal dilatation, intimal thickening, and vascular wall destruction with perivascular lymphohistiocytic infiltrate [5]. Vascular involvement in the form of cerebral [5] or carotid artery vasculitis with stenosis [6] has been also described in stroke in a patient with schistosomiasis (cases 5 and 8).

### 3.6. Watershed Infarction

Watershed infarction has been described in four of the cases reviewed here [6, 11, 12, 15]. Watershed infarcts involve the junction of the distal fields of two non-anastomosing arterial systems; in autopsy studies, they represent 10% of all brain infarcts. Being seldom fatal, the incidence is probably underestimated [20]. It is well established that severe systemic hypotension can cause bilateral watershed infarction in patients with carotid artery pathology, and watershed infarcts distal to internal carotid artery disease are more likely with a noncompetent circle of Willis [20], as in case 5. It is of interest that many of the reported cases suffered from additional cardiac complications that could have led to hypotension, including myocardial fibrosis, endocarditis, or arrhythmias (cases 1, 2, and 7).

### 3.7. Laboratory Investigations

Laboratory investigations used to diagnose schistosomiasis include optical microscopy, serological investigations, and PCR test. False-negative results at microscopy can occur in the first stages of investigations until the mature parasites start releasing eggs in the stool. Serological testing is a sensitive diagnostic method and also has high specificity (>95%), suitable for detecting low-grade infection but still not 100% sensitive: some cases of schistosomiasis may be detected by microscopy or PCR despite negative serology. This is also true for optical microscopy assessment: analysis of urine or stool samples will give negative results until the parasites are sexually mature and have started egg production: a definitive diagnosis therefore cannot be made until at least 12 weeks after the last exposure [21].

### 3.8. Relation between Cerebrovascular Events and Laboratory Findings

A positive optical microscopy investigation for Schistosoma spp. ova in the stool or urinary samples was reported in four patients. It is of interest that positivity occurred during follow-up, while the first assessment at hospitalization was reported to be negative. In case 1, optical Kato-Katz assessment of stool showed the presence of Schistosoma ova only after 3 weeks from hospitalization, when seroconversion occurred [11]. This also occurred in case 3, with a positive urinary sample 4 months after the neurological event [13], and in case 7, with watershed infarction occurring after a 2-week history of fever [15]. These findings can be explained by the fact that analysis of urine or stool samples may give negative results for the presence of ova until the parasites are sexually mature, as explained earlier [21].

### 3.9. Pharmacological Management and Side Effects

Steroids, heparin, and praziquantel have been used to treat neurological events in patients. Corticosteroids have been reported to be rapidly effective in the treatment of patients with stroke [6, 12, 13, 15, 16], intracranial vasculitis [6, 13], and in those patients with inflammatory complications occurred after the start of praziquantel therapy (see below) [13]. In this context, steroids might prevent endothelial cell damage by eosinophils [15]. Steroidal therapy has been used with success in patients with concomitant neurological and cardiological manifestations, as in the case of watershed infarction complicated by myocarditis [12].

Praziquantel can cause worsening neurological symptoms a few days after starting the treatment. This manifestation is possibly caused by schistosoma lysis and consequent immunological reaction. Clinical worsening after praziquantel treatment during acute schistosomiasis has been described in 40% of patients [15]. In case 5 of the present review, Praziquantel administration led to the exacerbation of carotid vasculitis, which was then effectively treated with intravenous methylprednisolone.

Camuset et al. report a rapid improvement in 28 years old patients with watershed infarct and carotid vasculitis with immediate treatment with anticoagulant and steroid therapy (prednisone 1 mg/kg/day). Praziquantel, added 1 week later, was tolerated in the first cycle, but induced worsening of vasculitis on the second cycle, 1 month later [6], successfully treated with methylprednisolone (500 mg/day) and switched to oral cortico-therapy (1 mg/kg/day) for 6 weeks.

Albendazole was initially started because of its wide spectrum in the 54 years old patient from Mali with cerebral vasculitis, with slow neurological improvement. In this case, seroconversion for schistosomiasis was only detected 4 months after the onset of symptoms, when praziquantel therapy was started. The patient was lost on follow-up [13].

Steroids treatment with prednisone 1 mg/kg was effective for the treatment of the 21 years old patient returning from Mali with acute schistosomiasis, which developed cerebral vasculitis 2 days after starting praziquantel therapy [13].

Prednisolone (2 mg/kg/day) tapered within 1 month also led to full clinical recovery within 5 days in the 19 years old patient who returned from Kenya, which manifested watershed infarction after acute schistosomiasis. This patient was additionally treated with praziquantel 4.8 mg on day 1, repeated after 7 days after schistosoma egg detection, which occurred 4 months after acute schistosomiasis first manifestations [22].

Praziquantel and aspirin therapy, but no steroids, was prescribed to the 25 years old patient who, after returning from Mali, presented with acute schistosomiasis followed by bilateral border-zone infarction after 1 week of fever. Concomitant progressive cardiomyopathy required surgery 16 months from onset [11].

### 3.10. Pharmacological Treatment: The Role of Steroidal Therapy

Corticosteroids have been reported to be rapidly effective in the treatment of patients with stroke and a history of schistosomiasis [6, 12, 13, 15, 16]. Steroidal therapy has been used with success in patients with associated neurological and cardiological involvement, such as those presenting myocarditis [12]. As reported by Grandière-Peréz and Caumes in their case report and discussed in the previous section of this paper, eosinophils are toxic to endothelial cells, and this toxicity may be impaired by methylprednisolone [15].

Interestingly, in the case reported by Sarazin et al., the 25 years old female patient with watershed infarction associated with progressive myocardial fibrosis finally requiring surgery, no steroidal treatment had been given [11]. Even though no conclusion can be drawn from analyzing the small number of cases, it can be speculated that steroidal therapy may have a role in arresting the progression of neurological and cardiac lesions secondary to the inflammatory burden of this specific condition.

### 3.11. Neurological Complications of Anti-Parasitic Drug: Praziquantel

Encephalopathy signs can occur a few days after a praziquantel treatment; schistosoma lysis with consequent immunological reaction can have a role in the occurrence of neurological manifestations of this anti-parasitic drug. Clinical worsening just after praziquantel treatment during acute schistosomiasis has been described in 40% of patients [15]. This was described by Camuset et al. in the 28 years old female patient returning from Burkina Faso with hemiplegia and language disorders, a junctional infarction, carotid stenosis, and contralateral carotid inflammation. Steroidal treatment was immediately started, and with the improvement of symptoms praziquantel therapy was started for schistosoma seropositivity and was initially well-tolerated, but 1 month later, the second course of praziquantel led to headache and diplopia, and sixth right cranial nerve paralysis, with new stenosis of the right carotid siphon diagnosed on magnetic resonance imaging. Exacerbation of carotid vasculitis was considered an adverse effect of praziquantel therapy, and resolution of the clinical picture, in this case, was acquired with intravenous methylprednisolone therapy [6].

## 4. Conclusions and Recommendations

Stroke might occur in patients with a history of schistosoma infestation, both acutely and in a chronic setting. The most frequent lesions are watershed infarctions. Carotid artery involvement in the form of narrowed lumen due to vasculitis may occur and have been reported in patients who were exposed up to 1 year earlier to the event, suggesting a role of chronic infestation. Clinicians should include schistosomiasis-related stroke in differential diagnosis, when assessing patients in endemic areas or travelers from those regions, given the importance of specific pharmacological management on patients' outcomes. Unfortunately, data available in the literature are limited to a small number of case reports, and are likely to be biased by understudying/underreporting of cases in endemic areas; therefore, definitive conclusions on various aspects of cerebral involvement in schistosomiasis are difficult to draw. More studies are needed to shed light on the cerebrovascular complications of schistosomiasis, following the newly-launched revised WHO 2021–2030 NTD Roadmap, which aims at the elimination of morbidity related to neglected tropical diseases in all endemic countries by 2030 [23].

## Figures and Tables

**Table 1:** Summary of case reports included in review.

Case number	Author	Age	Sex	Schistosomiasis details	Clinical presentation	Cerebrovascular involvement	Cardiac abnormalities	Schistosomiasis diagnosis	Additional events	Outcome
1	Sarazin 2004	25	F	Madagascar (3 months journey)	Day 0 = abdominal pain, diarrhea, myalgia. 1 week fever 38°C, rapidly progressive headache, limb ataxia, loss of memory	Bilateral watershed infarcts	Non-calcific endomiocardial fibrosis	Initial serology, stool and urine investigations negative. Stool positive and seroconversion after 3 weeks	Progression of cardiomyopathy in 16 months	No neurological recurrence

2	Sonneville 2006	56	F	n.a.	Day 0 = hemiplegia. History of recent progressive dyspnoea	Multiple watershed infarcts	Acute pericarditis and myocarditis	Positive serology	None	Neurological recovery at 6 month

3	Jaureguiberry 2007 pt 1	54	M	Lived in Mali for several years. Repeated exposure to fresh ground water	Day 0 = cerebellar syndrome and hemiparesis, confusion. 10 day history of fever 40°C	Multiple, bilateral cortical infarcts	ST tract inversion	Serology positive at 4 months	None	Psychomotor slowing and insomnia at 4 months

4	Jaureguiberry 2007 pt 2	21	M	Bathed in a lake in Mali 1 month before onset (maculopapular rash)	Day 0 = myalgia, headache, fever 39°C. Day 2 = started praziquantel. Day 4 = mental confusion, anosognosia	Cerebral vasculitis	Inverted *T* waves, high troponin	Positive serology, negative stool and urine	None	Full recovery in 48 h after steroid therapy

5	Camuset 2012	28	F	Burkina faso (mission-1 year), reported swimming in lake	Headache for 6 months Day 0 = transient hemiplegia, language disorders day1 = persistent hemiplegia	Watershed infarcts, carotid artery inflammation and stenosis	n.a	Positive serology, negative stool and urine, positive rectal biopsy	Development of VI cranial nerve palsy due to inflammation of carotid syphon, likely caused by praziquantel, 1 month after the first clinical observation	Minor improvement of carotid stenosis at 6 months

6	Wu 2012	n.a.	n.a.	n.a.	Acute headache, walking impairment	Multiple frontal lobes infarcts	None	n.a	n.a.	n.a.

7	Grandiere Peréz 2013	19	M	Bathed in Lake Victoria (Kenya) for 3 months	Day 0 = 15 days fever 39°C,weight loss, urticaria. Inverted T wave and positive troponin day 8 = recovery day 12 = ataxia, confusion, unilateral dysmetria	Watershed infarct	Myocarditis	Positive serology, negative stool and urine, then positive after 4 months	None	Full neurological recovery after a five-day steroid course

8	Nyein 2020	52	F	Visited hometown 1 year previously during schistosomiasis outbreak in Myanmar, ate snails, swam in pond	Day 0 = cognitive impairment, memory deficit, behavioral changes week 3 = dysarthria, hemiplegia, upper motor neuron facial palsy	Multiple cerebral infarcts, carotid and anterior cerebral arteries narrowing	None	Positive serology, negative stool + ova	New cerebral lesions after 3 and 9 weeks	Walked with aid at 6 months

“Day 0” refers to the first clinical evaluation. N.a: information not available.

**Table 2:** Anagraphic data and incidence of main clinical items. Values are expressed as mean ± standard deviation of the mean.

Anagraphic data	
Age	36.42 ± 16.7 (range 19–56)
Sex (*N* = 7)	F: 4 (50%) M: 3 (37.5%)

Clinical presentation	
Disorientation/confusion	5 (62.5%)
Cardiac abnormalities (myocarditis/ST or T abnormalities)	5 (62.5%)
Eosinophilia	6 (75%)
Fever	4 (50%)
Ataxia, gait impairment	4 (50%)
Hemiplegia	4 (50%)
Headache	3 (37.5%)
Urticaria	2 (25%)
Memory impairment	2 (25%)
Language impairment	2 (25%)

Cerebrovascular involvement	
Cortical lesions	3 (37.5%)
Cerebral vasculitis	3 (37.5%)
Watershed infarcts	5(62.5%)
External carotid artery involvement inflammation/stenosis	1 (12.5%)

Laboratory investigations	
Exclusion of other conditions	5 (62.5%)
Positive serology on admission	5(62.5%)
Negative stool and urine on day 0	5(62.5%)
Late stool and urine positivity	2(25%)

Neurological and cardiac additional events	
Additional events	3 (37.5%)

Neurological outcome	
Partial recovery	3 (37.5%)
Full recovery	4 (50%)
Not specified	1 (12.5%)

Overall outcome	
Alive	5 (62.5%)
No follow up available	3 (37.5%)

**Table 3:** Clinical presentation, cerebrovascular involvement, laboratory investigation results, additional events, neurological and overall outcome details in patients with stroke and schistosomiasis.

Clinical presentation
Eosinophilia was the most frequent finding, followed by cardiac abnormalities (myocarditis/ST wave inversion), disorientation and confusion, fever, gait ataxia, hemiplegia, headache, urticaria, memory impairment, dysarthria and other non-specified speech disturbance.

Cerebrovascular involvement
Watershed infarction, cerebral vasculitis, vasculitis and stenosis of the extracranial portion of the carotid artery have been described in different patients affected by schistosomiasis reported.

Laboratory investigations
On admission patient have presented with positive serology or seroconversion occurred during hospitalization. In some cases were stool and urinary samples were negative on admission, a late stool and urine positivity has be observed.

Additional events
Case 5: VI nerve palsy deemed to be caused by worsening of the vasculitis at the right carotid syphon level, with consequent compression of the nerve trunk [6].
Case 8: new cerebral infarcts, detected at MRI, 9 weeks after the first event [16].

Neurological outcome
Case 1: no recurrence of neurological symptoms was reported, but details of outcome were not provided.
Cases 2, 4, 7: A full neurological recovery was reported respectively after 6 months, 48 h and 5 days after steroid therapy.
Case 3: psychomotor impairment and insomnia persisted at 4 months.
Case 5: no information on clinical improvement was reported. Diplopia due to VI nerve palsy regressed 24 hours after steroid therapy, with no relapse. Minor improvement of carotid stenosis was reported at 6 month follow-up [6].
Case 6: no specified outcome
Case 8: partial recovery of gait occurred, which became possible without aid.

Overall outcome
No deaths have been reported among cases

## Data Availability

All data are included in the submitted text and tables.

## References

[1] McManus D. P., Dunne D. W., Sacko M., Utzinger J., Vennervald B. J., Zhou X. N. (2018). Schistosomiasis. *Nature Reviews Disease Primers*.

[2] Richter J., Bode J. G., Blondin D. (2015). Severe liver fibrosis caused by Schistosoma mansoni: management and treatment with a transjugular intrahepatic portosystemic shunt. *Lancet Infectious Diseases*.

[3] Abdelnaby M., Almaghraby A., Saleh Y., Ziada K. (2019). A bilharzial pulmonary artery aneurysm with a large calcified mural thrombus. *International Journal of Cardiovascular Imaging*.

[4] Ramadan M. M., Ramadan M. M. (2015). A huge thrombosed pulmonary artery aneurysm without pulmonary hypertension in a patient with hepatosplenic schistosomiasis. *American Journal of Case Reports*.

[5] Berkowitz A. L., Raibagkar P., Pritt B. S., Mateen F. J. (2015). Neurologic manifestations of the neglected tropical diseases. *Journal of the Neurological Sciences*.

[6] Camuset G., Wolff V., Marescaux C. (2012). Cerebral vasculitis associated with Schistosoma mansoni infection. *BMC Infectious Diseases*.

[7] Ezzati P. M. (2018). Health policy NCD countdown 2030: worldwide trends in non-communicable disease mortality and progress towards sustainable development goal target. *Lancet*.

[8] Adindu A. (2014). The need for effective management in african health systems. *Journal of Health Management*.

[9] Murad M. H., Sultan S., Haffar S., Bazerbachi F. (2018). Methodological quality and synthesis of case series and case reports. *BMJ Evidence-Based Medicine*.

[10] Behzadi P. G. M., Gajdacs M. (2021). Writing a strong scientific paper in medicine and the biomedical sciences: a checklist and recommendations for early career researchers. *Biologia Futura*.

[11] Sarazin M., Caumes E., Cohen A., Amarenco P. (2004). Multiple microembolic borderzone brain infarctions and endomyocardial fibrosis in idiopathic hypereosinophilic syndrome and in Schistosoma mansoni infestation. *Journal of Neurology Neurosurgery and Psychiatry*.

[12] Sonneville R., Lagrange M., Guidoux C. (2006). [The association of cardiac involvement and ischemic stroke in churg strauss syndrome]. *Revue Neurologique*.

[13] Jauréguiberry S., Ansart S., Perez L., Danis M., Bricaire F., Caumes E. (2007). Acute neuroschistosomiasis: two cases associated with cerebral vasculitis. *American Journal of Tropical Medicine and Hygiene*.

[14] Wu L., Wu M., Tian D. (2012). Clinical and imaging characteristics of cerebral schistosomiasis. *Cell Biochemistry and Biophysics*.

[15] Grandière-Pérez L., Caumes E. (2013). Corticosteroids for watershed infarction in acute schistosomiasis. *Clinical Infectious Diseases*.

[16] Nyein A. M., Sann A. A., Aye N. N., Tan C. T. (2020). Delayed onset cerebral vasculitis from chronic schistosoma mansoni infection in Myanmar: a case report. *Neurology Asia*.

[17] George M. G. (2020). Risk factors for ischemic stroke in younger adults a focused update. *Stroke*.

[18] Costain A. H., Macdonald A. S., Smits H. H. (2018). Schistosome egg migration: mechanisms, pathogenesis and host immune responses. *Frontiers in Immunology*.

[19] Nation C. S., Da’dara A. A., Marchant J. K., Skelly P. J. (2020). Schistosome migration in the definitive host. *PLoS Neglected Tropical Diseases*.

[20] Momjian-Mayor I., Baron J. C. (2005). The pathophysiology of watershed infarction in internal carotid artery disease: review of cerebral perfusion studies. *Stroke*.

[21] Kristiansen T., Pettersen F. O., Lier T., Hinderaker S. G., Greve G. M. K. (2021). Schistosomiasis in norwegian students after travel to Africa. *Tidsskr Nor Laegeforen*.

[22] Caumes E., Vidailhet M. (2010). Acute neuroschistosomiasis: a cerebral vasculitis to treat with corticosteroids not praziquantel. *Journal of Travel Medicine*.

[23] Mawa P. A., Kincaid-smith J., Tukahebwa E. M., Webster J. P., Wilson S. (2021). Schistosomiasis morbidity hotspots: roles of the human host , the parasite and their interface in the development of severe morbidity. *Frontiers in Immunology*.

